# Development of Vacuum-Chamber-Type Capacitive Micro-Pressure Sensors

**DOI:** 10.3390/mi16111290

**Published:** 2025-11-18

**Authors:** Lung-Jieh Yang, De-Yu Jiang, Wei-Chen Wang, Chandrashekhar Tasupalli, Horng-Yuan Shih, Yi-Jen Wang

**Affiliations:** 1 Department of Mechanical and Electromechanical Engineering, Tamkang University, New Taipei City 251301, Taiwan; 950284ddddd@gmail.com (D.-Y.J.); bmw001234567@gmail.com (W.-C.W.); chandrasekharamma25@gmail.com (C.T.); 2 Department of Electrical and Computer Engineering, Tamkang University, New Taipei City 251301, Taiwan; hyshih.tw@gmail.com; 3 Taiwan Semiconductor Research Institute, National Institutes of Applied Research, Hsinchu 300091, Taiwan; nick.wang@niar.org.tw

**Keywords:** capacitive pressure sensor, complementary metal oxide semiconductor (CMOS), vacuum chamber

## Abstract

This study presents the development of a capacitive pressure sensor tailored for measuring the dynamic pressure of flow fields. The sensor is fabricated using the UMC 0.18 μm CMOS-MEMS process, incorporated with additional post-processing steps such as metal wet etching, supercritical CO_2_ drying, and parylene encapsulation. The sensing architecture employs AD7746 as a capacitance-to-voltage converter (CVC), enabling the conversion of capacitance signals into voltage outputs for enhanced measurement fidelity. Structurally, the capacitive pressure sensor features a vacuum-sealed diaphragm capsule design with dual movable circular membranes functioning as sensing electrodes. A contact-mode capacitive configuration with a trapezoidal or Gong-like vacuum-chamber diaphragm is adopted to improve linearity and sensitivity. The output sensitivity was determined to be feasible for measuring dynamic pressure at 1–2 Pa resolution.

## 1. Introduction

The demand for micro-electro-mechanical system (MEMS) sensors is increasing annually to provide machines with more accurate predictive capabilities in areas such as industrial AI and energy efficiency technology [1,2]. Among these applications, flow and pressure sensors are some of the most commonly used components [3], and they are mounted on rotary or flying objects exposed to airflow to measure aerodynamic parameters for understanding and verifying the working efficiency of the fluidic machinery [4,5,6,7]. Compared to the flow sensor, the pressure sensor for measuring dynamic pressure is more difficult to implement, as even the minimum pressure resolution of the application scenario is barely on the scale of Pa (N/m^2^).

The development of pressure sensors has significantly expanded applications in automobiles and other industries since the advent of solid-state or silicon-based piezoresistive pressure sensors with an electrical signal for monitoring and control [8,9]. Moreover, due to their small and compact sensor size—without substantially interfering with the measured environment—on-site measurements with multiple silicon sensors that could be mounted on the object’s surface can be used to analyze data in real time, either wirelessly or remotely [4,5,6,7]. In such research, the sensors are expected to dynamically measure the output voltage induced by the object’s motion, and they can assist in estimating aerodynamic performance in wind tunnel ground testing or even at real working sites.

However, some inevitable issues present considerable difficulty with respect to the resolution of piezoresistive pressure sensors. On the one hand, sensitivity and linearity depend on their measurement range; on the other hand, the thermal effect inflicts excessively severe consequences on semiconductor sensors due to their intrinsic temperature drift characteristic. In the design practice, it is worth noting that sensitivity can be enhanced by thinning the pressure membrane, but the temperature compensation technique is needed for accurate pressure measurements [10]. In addition, the piezoresistive pressure sensor is only good at detecting static pressure, and it experiences difficulties when carrying out dynamic pressure measurements. The presence of airflow in dynamic pressure measurements causes a serious off-axis design issue, where the piezoresistive pressure sensor behaves similarly to a resistive thermal detector (RTD) flow sensor, resulting in a higher flow sensor output than the pressure sensor output [11,12].

Capacitive pressure sensors have made immense progress in mitigating the issue of thermal effects, and their capacitance varies at a higher rate than that of piezoresistive sensors [11,12]. These sensors feature a simple structure, but before the 1980s, capacitance measurement was challenging and limited within the pF range. Nevertheless, the complementary metal oxide semiconductor (CMOS) is currently a mature technology, providing significant support for both MEMS capacitive sensor fabrication and capacitance detection resolutions beyond fF.

CMOS—a technology that integrates analog and digital electronic circuits for programming, signal processing, and data storage on a silicon wafer monolithically—excels in ultra-low power consumption and size reduction down to nm according to Moore’s law, and it is the mainstream technology used in Ics. Piezoresistive pressure sensors can, of course, be made by the CMOS process as well. However, capacitive pressure sensors outperform piezoresistive pressure sensors in terms of the following: high sensitivity, low power consumption, and reduced susceptibility to thermal effects [13,14,15,16]. In other words, there is no doubt that CMOS capacitive pressure can be developed to detect dynamic pressure changes in the field of on-site measurement.

The authors of [17] propounded a capacitive pressure sensor with a double deformable diagram using CMOS MEMS fabrication. This study builds upon the operating principle of the touch-mode pressure sensor [18,19] and optimizes it into a more stereo configuration: a vacuum-chamber-type capacitive pressure sensor fabricated using CMOS and additional post-processing steps.

## 2. Materials and Methods

### 2.1. Conception Design

**Figure 1:**
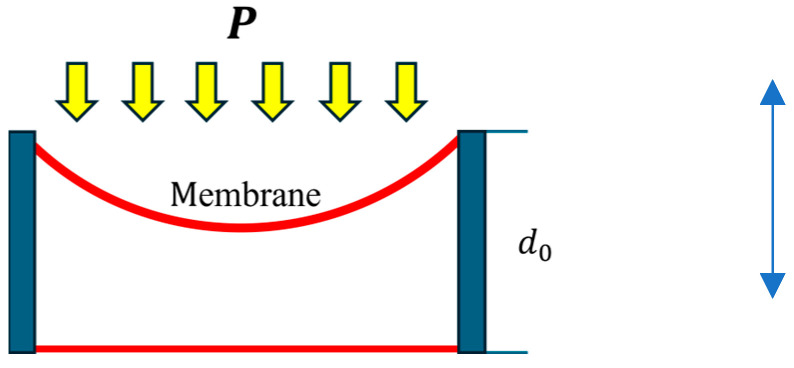
The operation of a capacitance sensor.

As shown in Figure 1, when a sensing electrode membrane deforms under pressure, the capacitance changes due to variations in the gap between parallel plates. By measuring the variation in capacitance, the change in pressure can be determined. In general, there is only one deformed membrane (the other side is the rigid substrate), and the capacitance changes nonlinearly with the gap according to the following equation:

(1) $$C=\varepsilon \frac{A}{d}$$

where ε is the dielectric constant, A is the area of two metal plates, and d is the distance between them. In addition, the deformation of membranes relates the geometric structure to a distributed pressure load.

**Figure 2:**
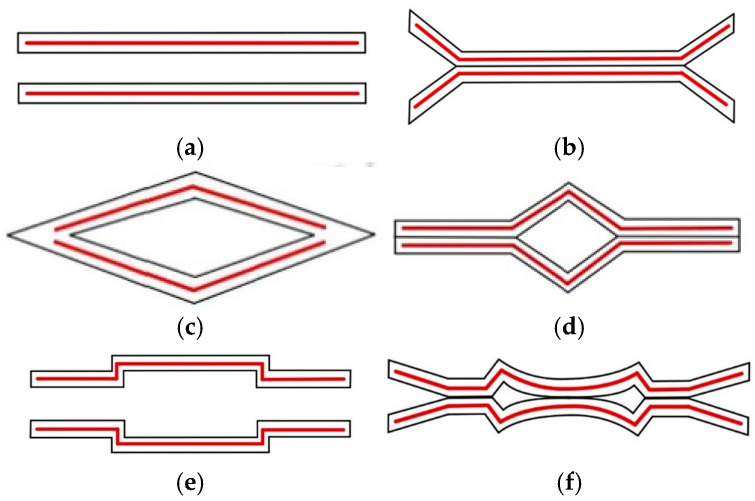
(**a**) Contact pressure membrane before loading; (**b**) contact pressure membrane after loading; (**c**) ideal new contact pressure membrane before loading; (**d**) ideal new contact pressure membrane after loading; (**e**) practical new pressure membrane with trapezoidal cross-section before loading; (**f**) practical new pressure membrane with trapezoidal cross-section after loading.

Before discussing improvements to the linearity of the pressure sensor, a double-deformable membrane configuration, or the vacuum-chamber geometry shown in Figure 2a, was preferred here to double the value of the output sensitivity of the sensor shown in Figure 1. The CMOS MEMS process makes this chamber’s fabrication easier [17].

Regarding the improvement in the output’s linearity, the touch-mode design exhibits its deformation (firstly contacted at the center) as shown in Figure 2b [18,19]. This design was invented to convert the gap-dominated behavior to area-dominated behavior to produce a more linear capacitance output. However, the linear output occurs within the high-pressure range and may cause damage at the membrane edge. Therefore, it requires modifications for low-pressure-range applications.

To prevent structural failure at the membrane edges, converting the square vacuum chamber shown in Figure 2a into the diamond shape shown in Figure 2c is a viable solution. Contact begins at the edge, as shown in Figure 2d, even under low-pressure loading. The corresponding deformation is shown in Figure 2d. However, the diamond chamber is three-dimensional (3D) and can only be approximated by stacking several CMOS layers to partially realize the 3D geometry in practice. Figure 2e shows the two-step approximation to the diamond chamber under the 6 metal layers provided by the 0.18 μm CMOS process, and Figure 2f shows the suspected deformation. According to the developing scenario of the CMOS process, more metal layers (e.g., 15 metal layers for a 3 nm node process) are available and will be beneficial for perfectly matching the 3D trapezoidal shape.

Figure 2b,d,f show two membranes coming into contact under pressure loading without experiencing sticking effects. This is because the membrane’s surface—after the supercritical CO_2_ treatment—becomes hydrophobic and self-cleansing, and they are not apt to stick to each other when in contact under pressure loading [20,21].

If the new capacitor design shown in Figure 2e is valid, the total capacitance before pressure loading can be expressed via Equation (2):

(2) $$C=C_{i}+C_{o}=\varepsilon \frac{\pi r^{2}}{3d}+\varepsilon \frac{\pi r^{2}(2^{2}-1^{2})}{d}$$

where the total capacitance, inner circle capacitance, and outer ring capacitance are *C*, $C_{i}$, and $C_{o}$, respectively; *r* and 2*r* are the radii of the inner circle and the outer ring, respectively; and *3d* and *d* are the capacitor gaps of the inner circle and the outer ring, respectively. Herein, the outer ring electrode $C_{o}$ dominates the total capacitance substantially more than $C_{i}$ at one order of magnitude.

### 2.2. CMOS MEMS Sensor Fabrication

MEMS is one of the methods within micro-scale mechanical fabrication, including processes such as chemical vapor deposition (CVD), anisotropic or sacrificial etching, wafer bonding, etc. This method is used to combine the electrical engineering systems applied in a wide range of fields with respect to electronics, mechanical engineering, materials science, optics, chemistry, and control systems. This study is focused on designing and fabricating a vacuum-chamber-type capacitive pressure sensor—in cooperation with the Taiwan Semiconductor Research Institute (TSRI)—using the UMC 0.18 μm CMOS MEMS process, with the suspended structure realized via a post-fabrication metal wet etching process. MEMS leverages device miniaturization to achieve substantial improvements in structural robustness, sensitivity performance, and cost efficiency. This technology is divided into two fabrication processes—bulk micro-machining and surface micro-machining. Therefore, the capacitive sensor was adopted as the dominant manufacturing surface micro-machining technology, and the vacuum chamber was subsequently released via wet etching.

**Figure 3:**
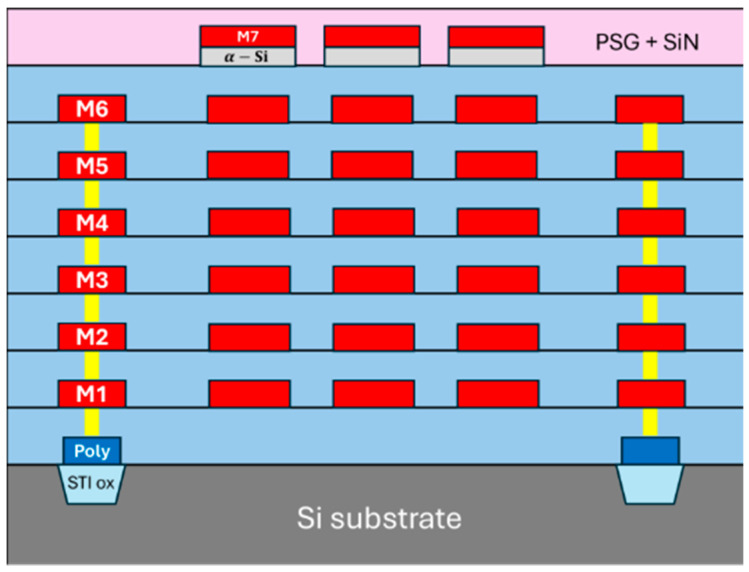
The standard of the CMOS process with respect to each layer.

The UMC 0.18 μm CMOS MEMS process combines CMOS with MEMS on chips using 0.18 μm technology nodes. It is composed of 1 poly-silicon layer, 5 inter-layer dielectrics (ILDs), 1 passivation layer, 6 layers of tungsten vias, and 6 metal layers (M1–M6). Phosphorous silicate glass (PSG) has multiple roles in the passivation layer of CMOS devices: It acts as a getter agent, a planarizing or smoothing layer, and a chemical barrier against moisture and other contaminants [22]. Shallow trench isolation (STI) oxide is used in CMOS fabrication to electrically isolate devices, and it provides better device isolation with reduced area consumption, while also ensuring a flat surface for advanced CMOS scaling and reliable circuit performance. A metal 7 (M7) layer was further introduced to serve as a masking layer with additional patterned amorphous silicon (α-Si) for isotropic etching. The illustration is shown in Figure 3.

### 2.3. Layout Design

**Figure 4:**
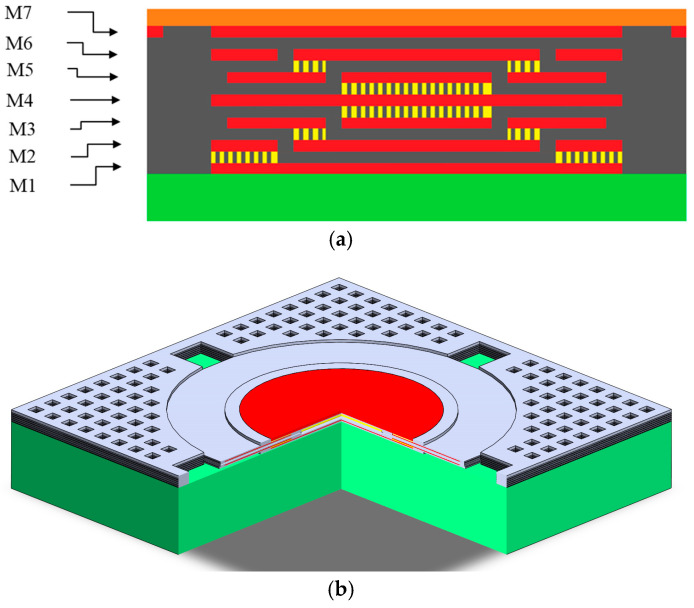
(**a**) The cross-section of the capacitive pressure sensor; (**b**) the capacitive pressure sensor in 3D vision with 4 larger openings (30 × 30 μm^2^), shown in green color.

**Figure 5:**
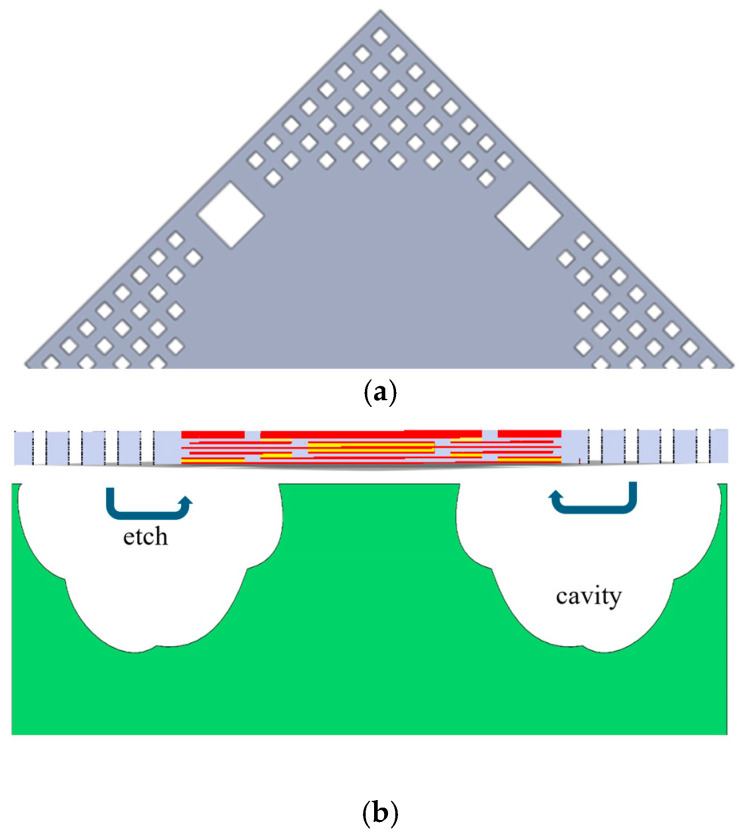
(**a**) Top view; (**b**) cross-sectional view of the vacuum chamber’s release from the substrate such that both sides of the vacuum chamber can be subject to pressure loading.

In Figure 2e, the double membranes of the two-step vacuum-type pressure sensor were designed using the CMOS layers in Figure 3. The cross-section of the entire capacitive sensor is depicted in Figure 4a. The upper membrane consists of M5 and M6 electrodes wrapped with ILDs, and the lower membrane similarly consists of M2 and M3 electrodes wrapped with ILDs. The center vacuum chamber is formed by M3-M4-M5 sacrificial layers, creating a two-step Gong-like capacitor gap between two membranes. Furthermore, some open areas were defined as spacers using sacrificial M1 and M2 layers at the bottom and M6 at the top to complete the vacuum chamber’s geometry. To facilitate sacrificial etching, two etch hole sizes are implemented, as shown in Figure 4b. Four larger openings (30 × 30 μm^2^) enable lateral etching for the effective removal of the sacrificial material for the vacuum chamber, whereas hundreds of smaller holes (8 × 8 μm^2^) are used for XeF_2_ etching to release the vacuum chamber from the substrate, as shown in Figure 5.

**Figure 6:**
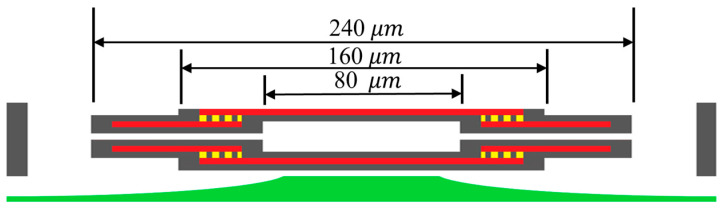
The dimension of the pressure sensor.

**Figure 7:**
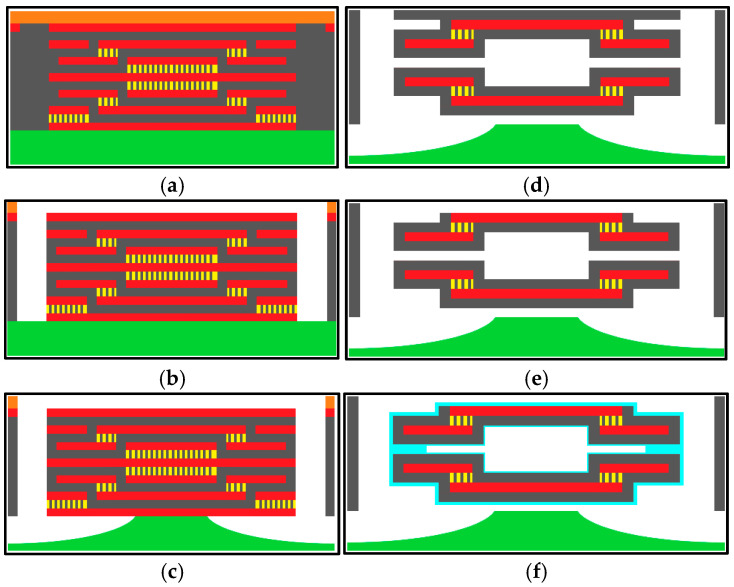
(**a**) The cross-sectional view of the layer; (**b**) the etching holes formed via anisotropic dry etching through the oxide layer; (**c**) the chamber on the silicon substrate using XeF_2_ dry etching; (**d**) sacrificial layer etching using the piranha solution [24]; (**e**) removal of the oxide layer using a laser cutting machine, packaging, and wire bonding; (**f**) vacuum sealing of the chamber via parylene coating.

Regarding the formation of the suspended membrane structure, packaging is employed to hermetically seal the vacuum chamber. This sealing process is crucial for preventing the presence of residual gases within the capacitive gap, as gas molecules inside the device may expand or contract with environmental temperature fluctuations, leading to variations in the gap and causing temperature-induced errors in the sensor’s output. In this study, polymer material deposition was adopted to seal the chamber using parylene-C. Parylene-C, with a relatively low Young’s modulus (E = 3.2 GPa) [23], ensures minimal influence on the mechanical behavior of the overall chamber structure. In addition, M3, M4, and M5, which serve as the primary Gong-like sacrificial layers for the vacuum chamber, are designed with four horizontal lateral etching channels connected to upward etching holes. These etching channels are located in the M4 layer, with an entrance dimension of 0.58 μm × 30 μm, ensuring effective vacuum chamber sealing during the 0.4 μm-thick parylene deposition. Furthermore, this deposition technique offers excellent step coverage, rendering it suitable for micro-scale encapsulation. The overall structural design dimensions are shown in Figure 6. Figure 7 summarizes the fabrication process, including the CMOS deposition and post-processes of XeF_2_, wet etching, and parylene coating.

### 2.4. COMSOL Simulation

**Figure 8:**
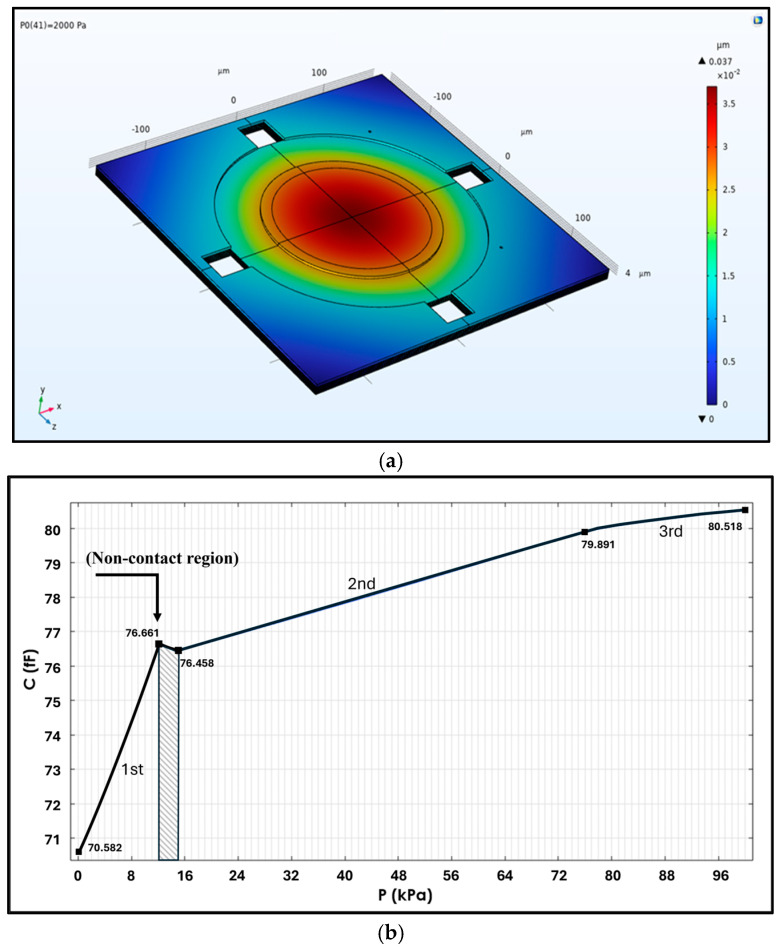
(**a**) COMSOL simulation; (**b**) capacitance vs. pressure of the vacuum chamber; the shadow inflection during 12–15 kPa denotes the first contact point’s inward shift.

COMSOL Multiphysics 6.3 is a simulation software based on numerical methods. Its “contact pair” is one of the functions that can simulate the contact behavior of the chamber-type capacitance (Figure 8a) within a certain range of pressure loading. Thus, the setup was applied under pressures ranging from 0 to 100 kPa with 500 computation nodes. The simulated capacitance vs. pressure result is shown in Figure 8b. From this capacitance change behavior, three distinct stages can be observed. The first stage (0–12 kPa) exhibits the highest sensitivity among the others, with a sensitivity of 0.5785 fF/kPa. This stage is primarily dominated by the deformation of the main membrane with some contact at the edge or the outer ring region, although its linearity is relatively lower than that of the second stage (15–76 kPa). The second stage is mainly attributed to the deformation of the central membrane, as the membrane’s edge ring has already fully contacted, leaving only the central membrane to undergo further deformation. Consequently, the sensitivity in this stage (0.0563 fF/kPa) is much lower than that in the first stage. Finally, the third stage, after 76 kPa, represents the saturation region. In this stage, either the ring edge or the membrane center enters touch mode, and the entire membrane chamber becomes very stiff. Its sensitivity of 0.0261 fF/kPa is also the smallest value of the three stages, as shown in Figure 8b.

### 2.5. Surface Stiction Issue

Surface stiction is caused by capillary forces generated by residual liquids remaining in the micro-structure after the etching process. This issue complicates MEMS structure design and reduces sensor quality [24,25]. The equation for evaluating the capillary Laplace pressure drop *P_c_* across a capacitor gap containing liquid is given by

(3) $$P_{c}=\frac{\gamma \cdot \cos\theta }{h}$$

where γ is the surface tension of the liquid (0.073 N/m for water), θ is the contact angle, and h is the small gap distance. As the gap distance is of the micro-meter range, the *P_c_* value often increases to the MPa scale, which is greater than the touch-mode pressure loading of 1 atm here. Therefore, surface stiction could not be avoided if this study did not adopt anti-stiction methods, e.g., the use of a CO_2_ supercritical dryer for liquid exchange and drying. The principle of supercritical drying is to replace the DI water with supercritical CO_2_, for which its surface tension γ is almost zero. When CO_2_ exceeds its critical point, the interface between liquid and gas disappears; there is no longer a clear distinction between the two phases, and surface tension is eliminated. In the supercritical drying process, the sensor die—after wet etching—is first immersed in DI water for cleaning and mass exchange. In the second stage, the DI water is replaced with isopropanol (IPA), which is compatible with supercritical CO_2_. The container holding the sensor die and IPA is then placed into the chamber of the supercritical dryer. After sealing the chamber, CO_2_ liquid is introduced and pressurized to the critical point. Once the CO_2_ drying process is complete, the chamber is opened, and the sensor die is retrieved, after which SEM can be used to inspect whether the membrane has experienced stiction.

## 3. Results

### 3.1. The Post-Etching Process

**Figure 9:**
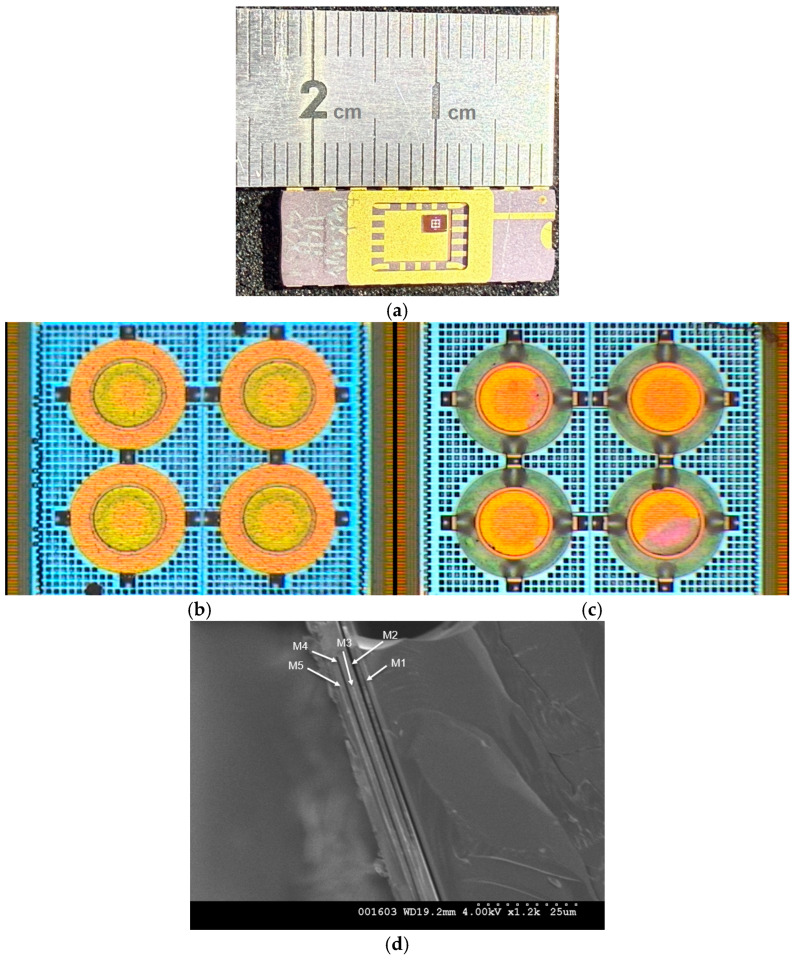
(**a**) The completed CMOS pressure sensor on the dual-in-line package (DIP); (**b**) the view of the capacitive pressure sensor after CMOS foundry and before etching; (**c**) the view of the capacitive pressure sensor via HCl etching; (**d**) the SEM image of the CMOS chip with the capacitor gap after etching.

The pressure sensor chip was etched using a 30 wt% HCl solution according to the wet etching process for 4 min. The metal layer containing M1, M2, M4, and M6 disappeared in anticipation of post-processing, as reflected in the CMOS chip shown in Figure 9a,b. Adhesion was observed in the green region of Figure 9b, causing the pressure to exhibit a self-sealing condition [26]. This phenomenon was confirmed during pressure testing, as no immediate leakage occurred and capacitance values could still be measured in time (it will be shown in the coming pressure testing section).

Since HCl hardly reacts with tungsten, it cannot completely etch either the via layers or the adjacent M3 and M5 layers. Consequently, the designated central circular sacrificial layers also cannot be completely removed, as shown in Figure 9c. As a result, the two-step Gong-like structure cannot be fully fabricated, preventing the implementation of the intended contact deformation. However, the M4 sacrificial layer can be removed to form the capacitor gap, exhibiting only conventional membrane deformations with a stiffened center boss. Therefore, the piranha solution, which is capable of etching tungsten vias, will be considered to replace HCl in future processes [24]. After completing the etching process, DI water and IPA were used to clean the sensor die, and the samples were placed into a CO_2_ supercritical dryer to free the sensor membrane. It was clearly observed that the ring portion of M6 had already been prematurely etched, and the green ring coloration at the center had disappeared, leaving only 64% of the electrode area intact. In addition, the SEM image in Figure 9d verified that there is no membrane collapse or stiction after wet etching and CO_2_ supercritical drying. The gap, approximately 0.61 µm, was etched from the original 0.65 µm thickness of the sacrificial M4 layer, which is nearly equal to the original design.

However, adding some dummy pressure chambers without metal electrodes alongside the real sensors is also recommended for evaluating the internal etching results via optical inspection in the future, similarly to the test keys or depth gauges commonly used in MEMS [27].

### 3.2. Small Capacitance Detection

In a capacitive pressure sensor measurement, it is necessary to regulate the ambient pressure and measure variations in capacitance. Excessive reliance on a data acquisition (DAQ) system with exposed wiring in the environment introduces significant parasitic capacitance, resulting in substantial interference in capacitance measurements. Therefore, this study adopts a capacitance-to-voltage converter (CVC), AD7746, to convert capacitance signals into voltage signals. AD7746 not only outputs voltage signals in terms of the corresponding capacitance value but also minimizes parasitic capacitance. The parasitic capacitance between the pressure sensor and the AD7746 circuit can be subsequently compensated, as outlined below.

For the system setup, AD7746 is first characterized without any cables to measure its parasitic capacitance and evaluate its measurement stability. Next, cables are connected for a second measurement, followed by connections to the PCB for a third measurement. This approach allows the pre-measurement of the total parasitic capacitance of all components, except for the sensor, ensuring the system’s stability. AD7746 exhibits an average capacitance of 2.344 fF, with root-mean-square (RMS) noise and peak-to-peak noise of 43 aF and 217 aF. After connecting the AD7746 to the cables and the PCB, the average measured capacitance increased by approximately 31 aF, which can be attributed to the inherent capacitance of the two coaxial cables. Stability was enhanced because the RMS noise and peak-to-peak noise decreased by 2 aF and 24 aF. This enhancement can be attributed to the shielding effectiveness of the cables, which mitigates electrostatic interference originating from the panel.

The PCB was designed using EasyEDA Pro v2.2.43.4 software, and the AD7746 chip allows the offsetting of parasitic capacitance via its accompanying software to identify the optimal circuit configuration. This capacitance measurement and analysis method effectively reduces parasitic capacitance and addresses resolution limitations.

### 3.3. Pressure Testing in a Vacuum Chamber

**Figure 10:**
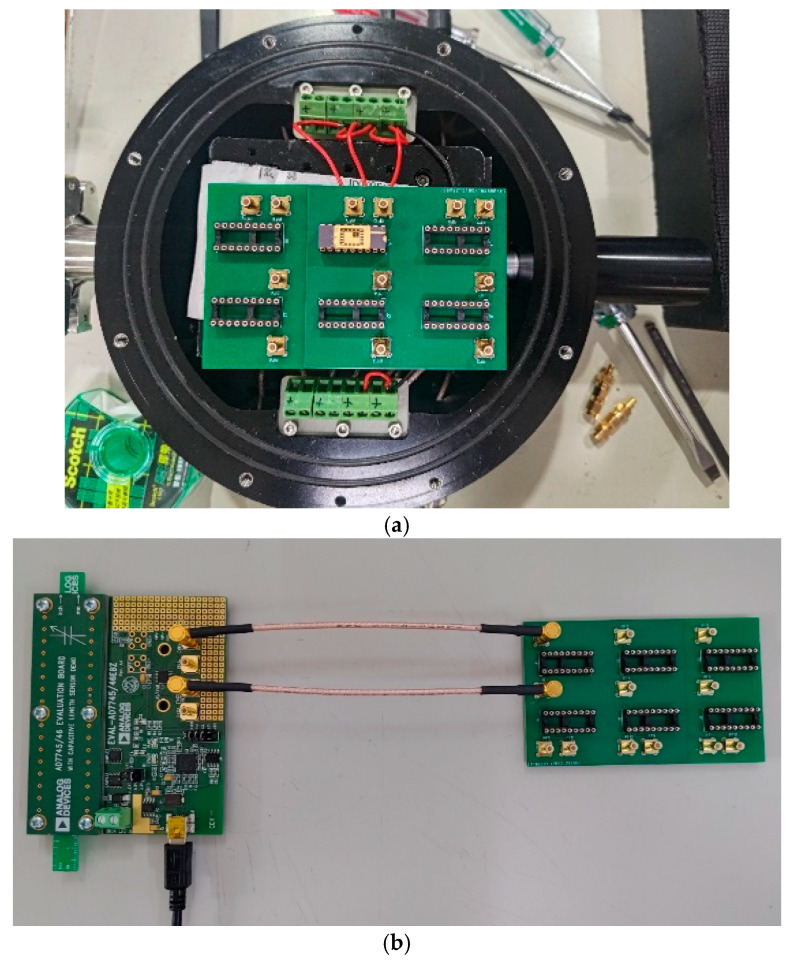
(**a**) The system of the vacuum vessel; (**b**) the arrangement of AD7746 with the PCB board.

The pressure testing of the vacuum-chamber-type pressure sensor was conducted in another vacuum vessel, as shown in Figure 10a, in order to simultaneously control environmental variations and analyze sensor signals. The CVC AD7746 in Figure 10b converts capacitance variations into voltage signals for subsequent processing. Since capacitance signals are more susceptible to factors such as cable length, diameter, spacing, and environmental electrostatics compared to voltage signals, AD7746 provides a more straightforward and direct method for measuring capacitance signals. The CVC is based on AD7746, which is a high-precision capacitance-to-digital converter (CDC) developed by Analog Devices. The output signals from the AD7746 were transmitted to analysis system, where DAQ and stability analysis were performed using the AD7746 v2.2 Evaluation Software.

**Figure 11:**
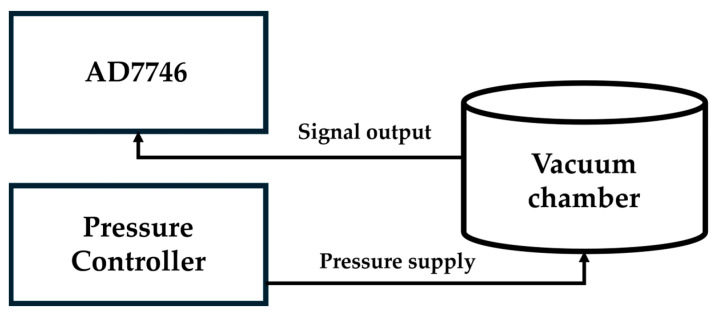
The schematic of the experimental setup.

The experimental setup of the vacuum vessel was in the range of 1 kPa to 100 kPa; this is within the range of low electrostatic interference, and the expected range of the resolution was higher than 16.5 bits. The experimental pressure testing and capacitance measurement setups are shown in Figure 11.

## 4. Discussion

**Figure 12:**
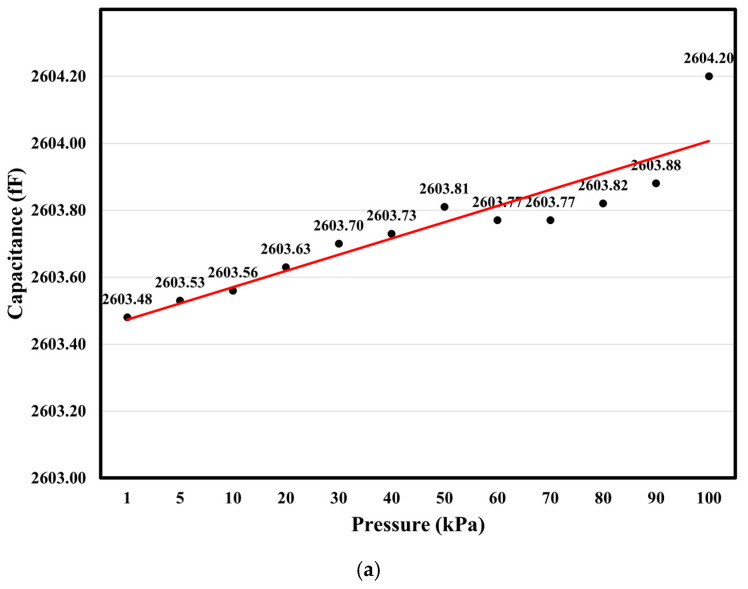

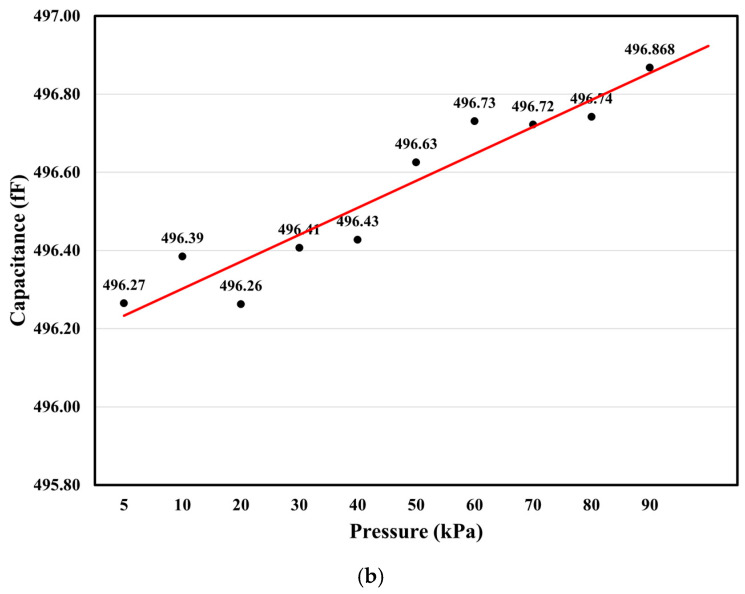
Measurement of capacitance versus pressure in a vacuum vessel with (**a**) the prototype capacitive pressure sensor and (**b**) the pressure sensor with parylene coatings.

The measurement results indicate that the device could still respond to applied pressure, suggesting that even after supercritical CO_2_ drying, deformations were concentrated in the peripheral annular structure due to the thicker, stiffened central boss on the membrane, which was more resistant to deformation [28]. The measurement results show that the average capacitance of the device at this stage, as shown in Figure 12a, was approximately 2603.8 fF, which is significantly higher than the originally designed and simulated capacitance value of approximately 320 fF (80 × 4 fF), as shown in Figure 8b.

The graph illustrates the linearity of capacitance over the range of 1 kPa to 100 kPa; this was calculated via linear regression, with a sensitivity of approximately 0.0051 fF/kPa. In the final stage, the capacitance change resembles the deformation shown in the simulation in Figure 8b, with a sensitivity of 0.0261 fF/kPa; however, it has dropped to only 19.6% of the originally predicted value.

In contrast, the average capacitance decreased to 496 fF when a 0.4 μm thick parylene coating was applied, as shown in Figure 12b. This may be due to the dielectric charging effect of parylene, which easily traps and holds electric charges on its surface [29]. Due to this material property, the electrostatic effect influenced the sensor’s behavior and the signal output of AD7746. Nevertheless, the capacitance changes generally aligned with expectations, with a sensitivity of approximately 0.0071 fF/kPa over the range from 5 kPa to 90 kPa. Data at 1 Pa and 100 kPa were excluded due to anomalies.

Regarding the capability of the developed capacitive pressure sensor to detect dynamic pressure, the evaluation is as follows. The simulation analysis in Figure 8b shows that, within the initial pressure range of 0–12 kPa, the maximum sensitivity reached 0.5785 fF/kPa, approximately 2.23 times higher than the reference value of 0.26 fF/kPa for a double-deformable-diaphragm capacitive pressure sensor. Moreover, the linearity of our new design demonstrated improved performance [17]. This implies that if the capacitance measurement module AD7746 achieves the best output resolution of 0.001 fF, then the minimum detectable pressure increment of this pressure sensor could be reduced to approximately 1.74 Pa. Consequently, the pressure sensor is capable of detecting the dynamic pressure increment corresponding to a minimum wind speed change of 1–2 m/s. However, given that the degenerated output sensitivity of the pressure sensor is only 0.0071 fF/kPa within the range from 5 kPa to 90 kPa, the minimum detectable dynamic pressure increment rises to 141.7 Pa, meaning that the sensor can only detect a minimum wind speed change of 16.8 m/s.

In addition, the effect of temperature on the completed capacitive pressure sensing module in this study was tested by increasing the temperature using a hot-plate heater. The sensor module was measured to assess the temperature effect. It was observed that the maximum capacitance difference between the sensor at a higher temperature (50 °C) and at room temperature (25 °C) was approximately 0.2 fF. The temperature coefficient (TC) is estimated to be approximately 25 ppm/°C. Compared to the pressure-induced capacitance output, the temperature-induced change of 0.2 fF is similar to the data fluctuations around the fitted output lines in Figure 12 and requires further investigation in the future.

## 5. Conclusions

The new vacuum-chamber-type capacitive pressure sensor, fabricated using the CMOS MEMS foundry and post-processing, demonstrates a maximum sensitivity of 0.5785 fF/kPa within an initial pressure range of 0–12 kPa. The sensor can detect a minimum pressure increment of 1.74 Pa and a minimum wind speed change of 1–2 m/s, rendering it suitable for on-site measurements of dynamic pressure and airflow speeds. Nevertheless, several improvements in the fabrication of the ideal vacuum-chamber-type capacitor structure are planned for the near future. These improvements include enabling the use of the piranha solution to remove metal sacrificial layers and tungsten vias after CMOS foundry processing to form the ideal two-step Gong-like capacitor configuration; adding dummy pressure chambers without metal electrodes alongside real sensors to facilitate optical inspection and evaluate internal etching conditions, similarly to the test keys or depth gauges commonly used in MEMS; and assessing potential damage to the pressure sensor’s membrane during the supercritical CO_2_ drying process due to its high pressure (73.8 bar). The remaining issues include considering the self-sealing of the pressure cavity through capillary adhesion to improve process flexibility and evaluating the dielectric charging effect of the parylene coating on sensor performance. With advantages such as compact size and reduced temperature effects, this CMOS capacitive sensor and actuator technology holds promise for future on-site applications in various unmanned vehicles operating in harsh environments.

## Data Availability

The original contributions presented in this study are included in the article. Further inquiries can be directed to the corresponding author.
